# Clinical examination remains more important than anorectal function tests to identify treatable conditions in women with constipation

**DOI:** 10.1007/s00192-012-1796-x

**Published:** 2012-05-23

**Authors:** T. J. Lam, R. J. F. Felt-Bersma

**Affiliations:** Department of Gastroenterology and Hepatology, VU University Medical Center, P.O. Box 7057, 1007 MB Amsterdam, The Netherlands

**Keywords:** Constipation, Rectocele, Anorectal manometry, Anal endosonography, Dyssynergia

## Abstract

**Introduction and hypothesis:**

Many women with chronic constipation are referred for anorectal function tests (AFT) when they fail initial conservative treatment with lifestyle advice and laxatives. Our goal was to prospectively investigate the diagnostic potential of AFT in women with constipation in order to identify treatable conditions.

**Methods:**

Between May 2003 and June 2011, all women with constipation referred to our tertiary referral center completed a questionnaire regarding their perianal complaints and underwent physical examination and were evaluated according to our AFT protocol, including anorectal manometry (ARM) and anal endosonography.

**Results:**

One hundred and thirteen women were referred and classified as having idiopathic constipation (*n* = 100), neurological disorder (*n* = 8), or others (*n* = 5). Of the 100 women with idiopathic constipation, clinical examination identified 25 (25 %) with hypertonia of the pelvic floor (dyssynergic pelvic floor) and 15 (15 %) with a rectocele. In 37/100 women also complaining of impaired evacuation, the yield of rectocele was 15 (41 %) and of hypertonia 5 (14 %). Women with hypertonia were younger (40 vs. 51 years; *P* = 0.002) and had no rectoceles identified (*P* = 0.02), and fewer women could relax during straining on ARM (56 % vs. 92 %; *P* < 0.001) compared with women without pelvic hypertonia. Other ARM measurements showed no differences between women with evacuation disorders, rectoceles, or hypertonia. Anal endosonography showed no internal sphincter hypertrophia.

**Conclusion:**

Potentially treatable conditions, such as rectocele and pelvic floor hypertonia, are found on clinical examination in 40 % of women with idiopathic constipation. Impaired evacuation is associated with the presence of a rectocele. AFT contributes little and should be reserved for selected cases.

## Introduction

Chronic constipation is a common disorder, with an estimated prevalence of 10–15 % and increases with age. Women are substantially more affected then men [1]. After excluding a colorectal tumour, lifestyle measures, laxatives, and sometimes physiotherapy are recommended to relieve the complaints of constipation, including abdominal fullness, hard stool, straining, and incomplete evacuation. Although many women respond to such a regime, a substantial number of women have ongoing complaints. Referral to specialized centers with anorectal testing facilities is often the next step. The thought behind this lies in the possible demonstration of underlying disorders requiring specific therapy. Rectocele, dyssynergic pelvic floor, or slow-transit constipation are generally thought to have different therapeutic approaches: a large rectocele requires surgery; a dyssynergic pelvic floor requires sustained physiotherapy; slow transit constipation requires high doses of laxatives, rectal cleansing, and—rarely—surgical intervention. It therefore seems important to recognize women with large rectoceles and/or a dyssynergic pelvic floor, as the presence of these conditions directly influences management.

A key question is which test(s) can discriminate between these diagnoses. Studies are not consistent regarding the value of anorectal function tests (AFTs) in patients with constipation. American Gastroenterological Association (AGA) guidelines and several studies recommend anorectal manometry (ARM) in patients with constipation [2–6]. However, other studies show that AFTs have no added value in patient management [7–10]. In our tertiary referral center, we perform ARM and anal endosonography (AUS) in patients referred for assessment of a possible underlying disorder. AUS can identify hypertrophy of the internal anal sphincter. We reserve defecography for patients with prolapse who are planned to undergo surgery but where there is uncertainty concerning the presence of an enterocele on clinical examination. Colon transit studies are reserved for patients with intractable constipation when colectomy is being considered. The purpose of this study was to critically evaluate the diagnostic yield of ARM and AUS in women referred with constipation.

## Patients and methods

The VU University Medical Centre (VUMC) is a tertiary referral center for anorectal function problems. Between May 2003 and June 2011, all women with constipation and who fulfilled the Rome III diagnostic criteria [11] were included. All women referred to us were prospectively evaluated by an extensive questionnaire regarding their perianal complaints; physical examination including abdominal, vaginal, and rectal examination; and our anorectal function protocol including ARM and AUS. All these data were entered into a database. In all of these women, secondary causes such as endocrine disorders or colonic obstruction were previously excluded by laboratory tests and colonoscopy. Women with inflammatory bowel disease, fissures, or fistulae were excluded.

Evacuation difficulties consisted of both difficulty in emptying as well as the feeling of incomplete evacuation. Women were considered to have a hypertonic pelvic floor or dyssynergia when there was a paradoxical increase in anal pressure or if they had great difficulty in relaxing or showed no relaxation at all during straining on three consecutive attempts, as assessed by one of the authors in a quiet setting in the presence of a female nurse. Only grade 2 or larger rectoceles (i.e., visible in the vulva during straining) were recorded as indicative of a rectocele. The questionnaire, physical examination, and AFT were performed for medical reasons. Therefore, the study did not require approval by the hospital’s commission of medical research ethics.

### Anorectal manometry

A four-microtip-transducer water-perfused catheter (Mui Scientific Type SR4B-5-0-0-0, Mississauga, Ontario, Canada) was used. The water-perfusion method was performed using a pull-through technique. With the patient lying in the left lateral position, the catheter was introduced into the rectum. After introduction, the catheter was withdrawn with the automatic puller at a speed of 1 mm/s to measure the maximum basal pressure (MBP) and the sphincter length (SL). The MBP was measured as the mean of the highest pressures at rest. The SL was the length over which the basal pressure was measured. Following this, the catheter was reintroduced and pulled back manually with steps of 0.5 cm to determine the maximum squeeze pressure (MSP) measured as the mean increase of pressure above the MBP during squeezing. The patient was then asked to strain on three separate occasions. No relaxation on straining was defined as no drop in pressure in the anal canal on straining under three consecutive attempts. Paradoxical contraction was defined as an increase in anal basal pressure upon straining.

The rectoanal inhibitory reflex (RAIR) was elicited by inflating the rectal balloon with the catheter positioned where the ARM was at highest. The volume necessary to inhibit MBP was recorded. Rectal compliance was measured with the use of the rectal balloon. Air was inflated manually using a syringe at a rate of 60 ml every 15 s. The volume of air needed to be inserted to illicit the first sensation of rectal distension, the urge to defecate, and the onset of intolerable distension similar to rectal capacity (RC), was measured.

### Anal endosonography

AUS was performed using a three-dimensional diagnostic US system (Hawk type 2050, B-K Medical, Naerum, Denmark) with a rotating endoprobe containing two crystals, covering 2-16 MHz (focal range 2–4.5 cm) (diameter 1.7 cm) producing a 360° view. During recording, the crystals were automatically pulled back by an internal puller, allowing longitudinal distances to be measured. More details of the AUS were published previously [12]. The integrity of the puborectalis muscle, external anal sphincter, internal anal sphincter, and submucosa were described.

### Statistical analysis

Results are presented as means and proportions. Fisher’s exact test and Pearson chi-square test were used to compare proportions, where appropriate. Student’s *t* test was used to compare differences in AFTs. Analyses were performed with the statistical software SPSS version 15.0.

## Results

In total, 113 women with constipation were referred. They were classified as having idiopathic constipation (*n* = 100), neurological disorder (*n* = 8), solitary rectal ulcer syndrome (SRUS) (*n* = 3), or Hirschsprung’s disease with surgery in childhood (*n* = 2). Patient characteristics are shown in Table 1 and AFT results are shown in Table 2.

**Table 1:** Clinical characteristics of patients with constipation

Cause of constipation	Idiopathic (*n* = 100)	Neurologic (*n* = 8)	*P* value
Age (year) (SD)	48 (16)	61 (14)	0.03
Duration constipation (years) (SD)	8.9 (7.8)	3.6 (4.2)	0.06
Evacuation problem (yes) (%)	37 (37)	3 (38)	NS
Operation in the past (yes) (%)	49 (49)	3 (38)	NS
Pelvic hypertonia (yes) (%)	25 (25)	3 (38)	NS
Rectocele (yes) (%)	15 (15)	0	NS
Defecation frequency per day (SD)^a^	0.9 (1.3)	0.9 (0.6)	NS

*SD* standard deviation, *NS* not significant

^a^With laxatives and/or manual maneuvers

**Table 2:** Anorectal function test results in patients with constipation

	Idiopathic	Neurological	*P* value
	(*n* = 100)	(*n* = 8)	
Anorectal manometry			
MBP (mmHg) (SD)	53 (18)	40 (8)	0.002
MSP (mmHg) (SD)	38 (19)	29 (15)	NS
SL (cm) (SD)	3.2 (1.0)	3.1 (0.9)	NS
Strain relaxation			
Yes (%)	83 (83)	5 (63)	NS
No (%)	13 (13)	2 (25)	
Paradoxical (%)	4 (4)	1 (13)	
RAIR			
Yes (%)	99 (99)	7 (88)	NS
No (%)	1 (1)	1 (13)	
RAIR (ml) (SD)	25 (12)	26 (10)	NS
FS (ml) (SD)	90 (55)	140 (76)	0.06
Urge (ml) (SD)	168 (79)	194 (80)	NS
MTV (ml) (SD)	235 (92)	264 (60)	NS
Anal endosonography			
Defect (%)	28 (28)	4 (50)	NS
Haemorrhoidal tissue (%)	25 (25)	0	NS
Atrophy (%)	13 (13)	0	NS
Internal sphincter hypertrophy (%)	0	0	NS

*SD* standard deviation, *MBP* maximum basal pressure, *MSP* maximum squeeze pressure, *SL* sphincter length, *RAIR* rectoanal inhibitory reflex, *FS* first sensation volume, *MTV* maximum tolerable volume, *NS* not significant

### Idiopathic constipation

**Fig. 1:**
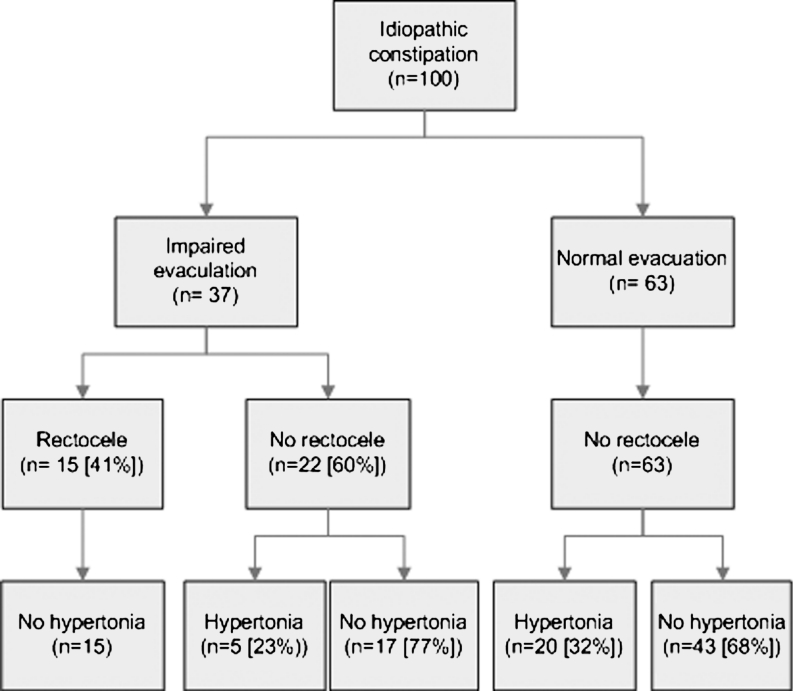
Idiopathic constipation and the presence of rectocele and pelvic hypertonia in female patients

One hundred women had idiopathic constipation (Fig. 1), of whom 37 (37 %) complained of incomplete evacuation. Of the 37 women with an impaired evacuation, clinical assessment identified a rectocele in 15 (41 %), hypertonia in five (14 %), and neither impaired defecation nor rectocele in 17 (45 %) (Table 3). No rectocele was present in the 63 women who experienced normal evacuation, but hypertonia was identified in 20 (32 %) of them. Fewer women with incomplete evacuation had coexisting anal hypertonia than women without (14 % vs 32 %; *P* = 0.05). There were no significant differences in ARM measurements between women with and without incomplete evacuation.

**Table 3:** Patients with and without impaired evacuation

	Impaired evacuation (*n* = 37)	No impaired evacuation (*n* = 63)	*P* value
Age (year) (SD)	53 (16)	45 (15)	0.02
Rectocele (yes) (%)	15 (41)	0	< 0.001
Hypertonia (yes) (%)	5 (14)	20 (32)	0.05
No relaxing during straining on ARM (%)	3 (8)	14 (22)	0.10
Other ARM measurement			NS

*SD* standard deviation, *ARM* anorectal manometry, *NS* not significant

Total hypertonia was identified in 25 women (25 %), five of whom (20 %) indicated impaired defecation. Women with pelvic hypertonia were significantly younger (40 vs. 51 years; *P* = 0.002) and had no rectoceles identified (*P* = 0.02), and more women could not relax during straining as reflected by ARM measurements (56 % vs 92 %; *P* < 0.001) compared with women without hypertonia (Table 4). ARM had a sensitivity and specificity for identifying pelvic floor hypertonia of 44 % and 92 %, respectively. No other significant differences in ARM parameters were found between women with and without hypertonia.

**Table 4:** Patients with and without pelvic floor hypertonia

	Hypertonia (*n* = 25)	No hypertonia (*n* = 75)	*P* value
Age (year) (SD)	40 (14)	51 (15)	0.002
Impaired evacuation (yes) (%)	5 (20)	32 (43)	0.056
Rectocele (yes) (%)	0	15 (20)	0.02
Relaxing during straining on ARM (yes) (%)	14 (56)	69 (92)	<0.001
Other ARM measurement			NS

*SD* standard deviation, *ARM* anorectal manometry, *NS* not significant

There were no significant differences in ARM measurements found between women with and without a rectocele. Women with a rectocele tended to have a higher stool frequency than those without (1.4 defecations/day vs 0.8 defecations/day with laxatives and/or manual maneuvers; *P* = 0.07). AUS showed anal sphincter defects (*n* = 28), hemorrhoidal tissue (*n* = 25), and atrophy of the anal sphincters (*n* = 13). No hypertrophy of the internal sphincter was detected.

### Neurological disorder

Eight women had constipation due to neurological disorders: cauda equina syndrome (*n* = 4), multiple sclerosis (*n* = 2), diabetes mellitus (*n* = 1), and a combination of diabetes mellitus and Parkinson’s disease (*n* = 1). Three women (38 %) had previously undergone surgery, and three women (38 %) had an evacuation problem. None of these women had a rectocele.

### Idiopathic constipation vs neurological disorder

Women with idiopathic constipation were slightly younger (48 vs 61 years; *P* = 0.03) and tended to have higher rectal sensitivity (i.e., lower volume to feel the balloon) (90 vs. 140 ml; *P* = 0.06),than women with neurological constipation.

## Discussion

This prospective study showed that ARM and AUS has little additional value in the evaluation of women referred with idiopathic constipation. On ARM, more women with a hypertonic pelvic floor failed to relax during straining than women without pelvic floor hypertonia (56 % vs 92 %). Thus, 56 % of women with a hypertonic pelvic floor demonstrated relaxation during ARM. This is understandable, as even women with a hypertonic pelvic floor will eventually pass some faeces, which is comparable with women with a hypertonic pelvic floor who can expel the balloon [7]. On the other hand, some women with a normal or low pelvic floor tone may have an inefficient way of expelling their hard faeces, thus mimicking dyssynergia with ARM. Another important finding was that hypertonic pelvic floor was not present in any of the women with a rectocele. Although this finding does not exclude the possibility that this combination ever occurs, it does emphasize the different pathogenesis of these two disorders. All patients were evaluated by inspection of the perianal area to identify the presence of a rectocele. After inspection, rectal examination was performed to identify pelvic floor hypertonia. Therefore, it is possible that the diagnosis of pelvic floor hypertonia occurred less often after identifying a rectocele. Although we cannot rule out this bias, patients were carefully examined and the total procedure took 1 h.

The pathogenesis of pelvic floor hypertonia is not completely understood but is probably multifactorial. It is often an acquired consequence of faulty toilet habit, obstetric or back injury, and brain–gut dysfunction [13]. Pelvic floor hypertonia has also been associated with sexual abuse in childhood [14]. This needs to be taken into account when determining overall management. A history of childhood sexual and physical abuse may be associated with diminished improvement after biofeedback for pain severity and mental health quality of life [15].

In our study, of the women who had an evacuation disorder, 41 % had a rectocele. No rectoceles were found in women without evacuation disorders. This implies that in women with evacuation disorders there is a large chance of finding a rectocele, and therefore, careful examination of pelvic floor may avoid further investigations. An interesting finding was that in women who stated that they had no evacuation difficulty, 32 % of them had a hypertonic pelvic floor on clinical examination and were thus unaware of the relationship between their constipation and their pelvic floor. This may explain the results of earlier studies that demonstrated that biofeedback has a positive effect in women with slow-transit constipation [16–18]. Hypertonia may still have been present, with normalization with biofeedback being responsible for the improvement.

Regarding the relationship between physical examination including digital examination and AFTs, there is a significant correlation between digital and manometric assessment of anal sphincter tone [19, 20]. For dyssynergia it is difficult to choose a gold standard between ARM and defecography. Both methods have been suggested as a gold standard in an attempt to objectively assess this straining disorder. Clinical examination by an experienced investigator is just as valuable [20]. In our study, ARM had a sensitivity and specificity for identifying pelvic floor hypertonia of 44 % and 92 % respectively, defined by digital rectal examination. On the other hand, digital rectal examination had a sensitivity and specificity for identifying dyssynergia of 75 % and 87 %, respectively, defined by using a combination of ARM, rectal balloon expulsion, and defecography [20]. However, digital examination may overdiagnose dyssynergia, as in 20 % of asymptomatic patients, the anal sphincter or puborectalis may not relax during defecation [21]. This observation is also seen using ARM. Although ARM and defecography have increased the insight into this defecation disorder, the diagnosis of dyssynergia can be very well made by clinical investigation, thus avoiding invasive tests and radiation exposure.

The normal variation of anorectal physiology is wide, and there is a considerable overlap between patients and controls. Abnormalities can, however, sometimes be found in patients with constipation. As mentioned earlier, nonrelaxation during straining can be observed in patients with dyssynergic or obstructed defecation [22]. Furthermore, low anal basal pressure and impaired rectal sensation has been described in patients with constipation [4, 19]. Although we agree that ARM provides additional information, patient management is not influenced.

In large rectoceles, an increased RC and compliance compared with small rectoceles has been described [23]. We found no difference in ARM measurements between women with and without a rectocele. A possible explanation is that in many patients with longstanding constipation, the rectum has already dilated and thus has increased RC. A rectocele can also be diagnosed with defecography, which can visualize evacuation and reveal the size of the rectocele. However, there is much variation among radiologists with respect to interpreting the results. In addition, the degree of prolapse does not always correlate with symptoms [8, 24]. Furthermore, digital examination can perfectly establish the presence of a rectocele [25]. For clinical management, only women with a large rectocele in combination with symptoms are eligible for surgery.

Although there are some differences in ARM findings between women with idiopathic and neurological constipation, ARM has no discriminatory value. Therefore, medical history remains most important. ARM is useful in ruling out Hirschsprung’s disease. Absence of the rectoanal inhibition reflex in combination with high anal basal pressure is pathognomonic for Hirschsprung’s disease in children. Adult-onset Hirschsprung’s disease is extremely rare and is accompanied by many other features, and subsequent surgery is generally successful [26]. Internal anal sphincter hypertrophia, a thickening of the internal sphincter, is a very rare cause of chronic constipation [27]. Apart from constipation that starts in childhood, there is a high anal sphincter tone. AUS can demonstrate this and should be performed in selected cases in which there is a high suspicion. However, routine AUS in patients with chronic constipation is not useful. Patients eligible for (sub)total colectomy should be evaluated by ARM, which allows an objective measurement of anal pressures and RC. It is important to be informed about the continence reserve in order to avoid fecal incontinence after surgery [28].

In conclusion, careful clinical examination remains pivotal in demonstrating a rectocele or pelvic floor hypertonia. ARM and AUS are not justified in the routine workup of women with chronic constipation. In the current financial climate, with cutbacks in healthcare spending, the judicious use of technology is an important issue. Additional testing should be reserved for selected cases with constipation from childhood or for patients in whom a (sub)total colectomy is being considered.
